# Unveiling the Silent Sinus Syndrome: A Familial Case Series Exploring Chronic Maxillary Atelectasis

**DOI:** 10.7759/cureus.77001

**Published:** 2025-01-06

**Authors:** Giovanny E Perez, Antonio Bures, Ana L Melero-Pardo, Francisco Garraton, Gabriel A Hernández-Herrera, Juan C Portela-Arraiza

**Affiliations:** 1 Otolaryngology - Head and Neck Surgery, University of Puerto Rico, Medical Sciences Campus, San Juan, PRI; 2 Otolaryngology - Head and Neck Surgery, University of Puerto Rico School of Medicine, San Juan, PRI; 3 Medicine, Universidad Central del Caribe, Bayamón, PRI; 4 School of Medicine, University of Puerto Rico, Medical Sciences Campus, San Juan, PRI

**Keywords:** chronic maxillary atelectasis, genetic basis, otolaryngology-head and neck surgery, rhinology diagnostics, silent sinus syndrome, spontaneous enophthalmos, surgical management

## Abstract

Chronic maxillary atelectasis (CMA) is characterized by a persistent reduction in maxillary sinus volume, resulting from inward bowing of its antral walls. Silent sinus syndrome (SSS), a rare manifestation of CMA, typically presents with significant bony structural changes but without the usual nasal symptoms. The primary clinical presentation of SSS often includes enophthalmos, which may lead to referrals to ophthalmology due to perceived ocular asymmetry. This case series presents the familial occurrence of CMA and SSS in three male family members, each exhibiting different degrees of nasal symptoms and imaging findings consistent with maxillary sinus opacification and collapse. The familial clustering of these cases suggests a possible genetic or hereditary component to the development of CMA and SSS, an aspect not commonly explored in the existing literature.

## Introduction

Spontaneous enophthalmos, often associated with a contracted ipsilateral maxillary sinus, is characterized by chronic maxillary atelectasis (CMA) and silent sinus syndrome (SSS). Although traditionally considered separate conditions, recent discussions suggest that SSS may represent a specific stage within the broader continuum of CMA [1,2]. CMA is typically classified into three progressive clinical-radiological stages: stage I, membranous deformity; stage II, bony deformity; and stage III, clinical deformity [3]. While sinonasal symptoms are always present in CMA, SSS is specifically used when this condition manifests with enophthalmos, hypoglobus, and, rarely, midfacial deformity, without the usual complaints related to nasal obstruction or sinusitis. Significant structural changes in the maxillary sinus, particularly involving the orbital floor, can lead to complications such as enophthalmos, hypoglobus, and midfacial deformities. These occur due to downward displacement of the orbital floor, which compromises eye support and alters both facial aesthetics and visual function [4].

The primary clinical manifestation of SSS is enophthalmos, which develops insidiously over weeks or months. Patients often seek medical evaluation due to noticeable eye asymmetry, prompting referrals to ophthalmologists. This asymmetry can be misinterpreted for exophthalmos of the contralateral eye, ptosis of the affected side, or enophthalmos of unknown origin. When more common causes of enophthalmos, such as metabolic, ocular, or neurological conditions, are excluded, imaging studies become crucial. A CT scan of the orbit and sinus generally reveals unilateral maxillary sinus opacification, collapse, and inferior bowing of the orbital floor.

Notably, SSS presents without typical sinus symptoms or a history of rhinosinusitis, and the condition is discovered incidentally during imaging. While it is termed “silent,” some patients may present with mild or vague nasal symptoms. Montgomery first identified this condition in 1964; since then, approximately 100 cases have been reported in the literature [5]. Early management of SSS, before 1993, often involved a Caldwell-Luc approach along with transconjunctival orbital floor repair. However, in 1993, Blackwell and colleagues introduced an important advancement, endoscopic maxillary antrostomy combined with transconjunctival orbital floor repair [4]. Their method proved effective, improving patient outcomes, including resolution of maxillary disease and preventing enophthalmos recurrence, as evidenced by follow-up evaluations and CT imaging. This surgical approach, with or without concurrent orbital floor reconstruction, remains a cornerstone in the management of SSS [6,7].

Although CMA and SSS are rare, the literature primarily focuses on characterizing clinical presentation, radiographic findings, treatment options, and outcomes. Risk factors for these conditions include chronic maxillary sinus obstruction, prior sinus surgeries, nasal anatomical variations, and possibly even familial genetic predispositions, though these are still under investigation. There remains, however, a paucity of reports on the genetic aspects of CMA and SSS. This case series aims to describe CMA and SSS within a family, offering insights into their potential genetic basis and encouraging further investigation into this aspect. Further investigation into the genetic components of CMA and SSS could enhance patient management and lead to more targeted, effective treatment strategies.

## Case presentation

Case 1 (father)

**Figure 1:**
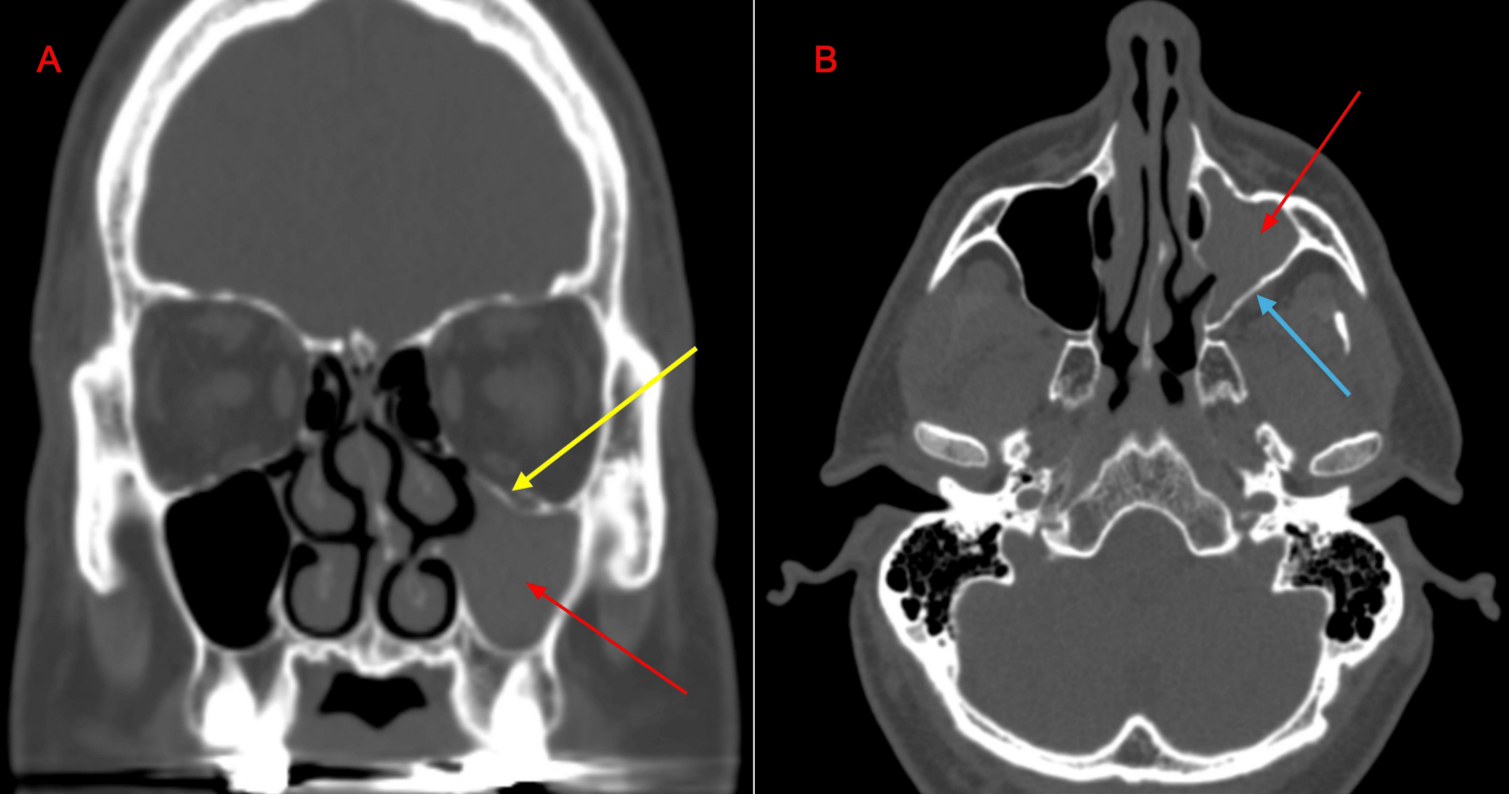
Non-contrast CT imaging of the paranasal sinuses showing left maxillary sinus opacification and associated structural changes. Coronal (A) and axial (B) views of a non-contrast CT scan of the paranasal sinuses demonstrating complete opacification of the left maxillary sinus (red arrows). Additional findings include atelectasis of the left uncinate process, bowing of the orbital floor (yellow arrows), and inward bowing of the posterior wall of the maxillary sinus (light blue arrow).

A 48-year-old male presented with nasal congestion with a Nasal Obstruction Symptom Evaluation (NOSE) score of 85 out of 100, persisting for eight months [8]. He reported a normal sense of smell and denied experiencing discolored nasal discharge or recent trauma. Physical examination was unremarkable. An endoscopic nasal evaluation revealed a deviation of the nasal septum to the left, a posterior septal deviation with a spur contacting the left lateral nasal wall, and moderate edema of the inferior turbinates, characterized by boggy and bluish mucosa with polypoid degeneration. A non-contrast CT scan of the sinuses demonstrated complete opacification of the left maxillary sinus, atelectasis of the left uncinate process, bowing of the orbital floor, and inward displacement of the posterior wall of the maxillary sinus, consistent with left maxillary SSS (Figure 1). The patient subsequently underwent a septoplasty for obstructive symptoms and a left maxillary sinusotomy and a partial left ethmoidectomy for the SSS changes. At a three-year postoperative follow-up, the patient reported substantial improvement in symptoms. Additionally, the patient subsequently brought both of his sons to the otolaryngology clinic for evaluation.

Case 2 (older son)

**Figure 2:**
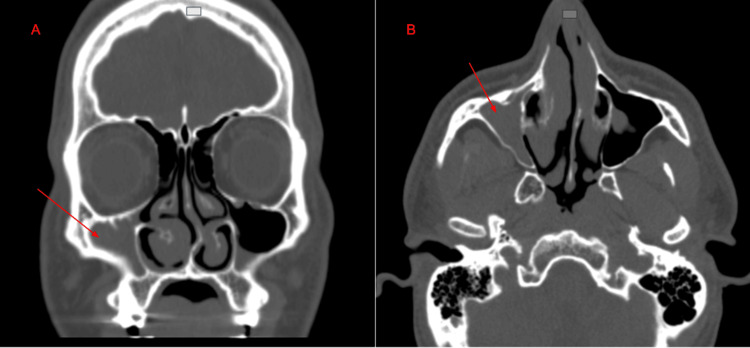
Non-contrast CT imaging of the paranasal sinuses revealing right maxillary sinus opacification and associated findings. Coronal (A) and axial (B) views of a non-contrast CT scan of the paranasal sinuses showing complete opacification of the right maxillary sinus (red arrows). Additional findings include atelectasis of the right uncinate process and inward bowing of the medial maxillary sinus wall.

A 22-year-old male presented with recurrent clear anterior nasal discharge and other symptoms consistent with allergic rhinitis. Physical examination, including inspection of the head, face, and ocular motility, showed no abnormalities. Endoscopic nasal evaluation revealed a severe left septal deviation both anteriorly and posteriorly, bilateral boggy and pale turbinate mucosa with a nodular appearance, and a convex caudal quadrangular cartilage. Additionally, there was a wide right middle meatus with atelectatic changes. The NOSE score was 37.5 on a scale of 100, and the Sinonasal Outcome Test (SNOT-22) score was 28.2 on a score of 0 to 110 [9]. Non-contrast maxillofacial CT scan indicated complete opacification of the right maxillary sinus, consistent with right CMA stage 1 (Figure 2). The patient underwent an endoscopic right maxillary antrostomy and a right partial ethmoidectomy. Postoperative follow-up demonstrated symptomatic improvement.

Case 3 (younger son)

**Figure 3:**
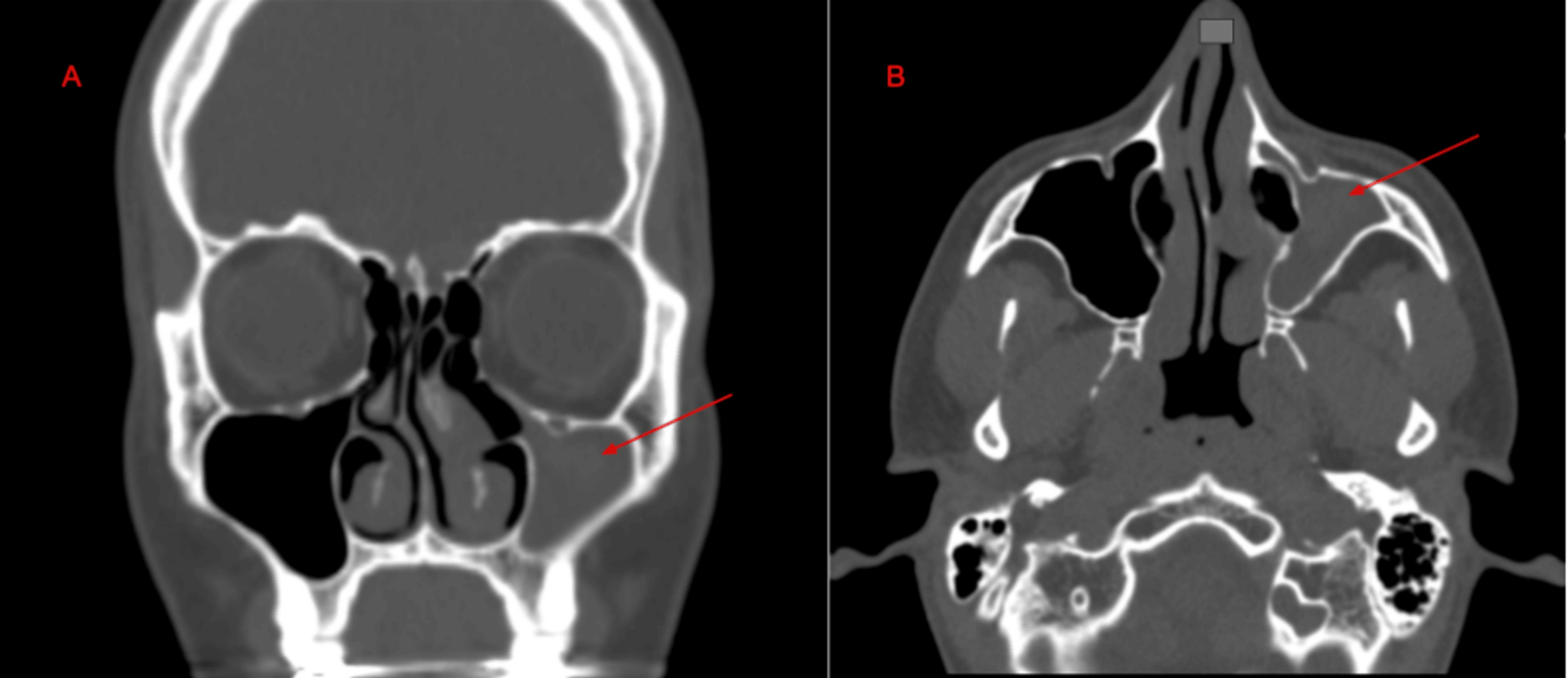
Non-contrast CT imaging of the paranasal sinuses demonstrating left maxillary sinus opacification and associated findings. Coronal (A) and axial (B) views of a non-contrast CT scan of the paranasal sinuses showing complete opacification of the left maxillary sinus (red arrows). Additional findings include atelectasis of the left uncinate process and inward bowing of the medial maxillary sinus wall.

A 20-year-old male presented with a chronic sore throat, moderate nasal congestion, and discolored postnasal drip. Physical examination was unremarkable. An endoscopic nasal evaluation revealed a right septal deviation, boggy and bluish turbinate mucosa, and polypoid edema of the left middle turbinate. A thick yellow discharge was observed exiting the middle meatus. The NOSE score was 25 on a scale of 100, and the SNOT-22 score was 25.4 on a score of 0 to 110. The patient was given adequate medical treatment for his chronic sinusitis. On follow-up, the patient continued with similar symptoms. A non-contrast CT scan of the sinuses showed complete opacification of the left maxillary sinus, consistent with left CMA stage 1 (Figure 3). The patient underwent a septoplasty to treat the obstructive symptoms, and an endoscopic maxillary antrostomy, anterior ethmoidectomy, and frontal sinusotomy of the left side to address the CT findings. Postoperative follow-up demonstrated symptomatic improvement.

## Discussion

The pathophysiology of SSS remains a topic of ongoing debate, but current theories suggest that sinus hypoventilation, caused by ostial obstruction and resulting negative pressure, plays a central role [3,10-12]. Patients typically present with spontaneous enophthalmos over the course of weeks to months, but often without significant sinus-related symptoms [10-12]. While clinical signs may raise suspicion for SSS, a definitive diagnosis is best achieved through radiologic imaging. Key imaging features of SSS include maxillary sinus opacification, collapse, and inferior bowing of the orbital floor; however, some patients may lack these pathognomonic findings [10,13,14]. SSS can be considered an end-stage manifestation of CMA. In CMA, the maxillary sinus progressively becomes smaller and less aerated, typically due to obstruction of the sinus ostium from causes such as chronic sinusitis, nasal polyps, or anatomical abnormalities. Over time, the lack of normal sinus ventilation and drainage may lead to the “silent” presentation of SSS. Although the cases in this series did not precisely match the classic profile of SSS, characterized by enophthalmos without prominent nasal symptoms, they highlight related aspects of CMA. The insidious onset of SSS, marked more by ocular changes than overt sinus symptoms, often complicates timely diagnosis, underscoring the importance of detailed imaging for accurate identification.

These cases underscore the necessity of including SSS in differential diagnoses when patients present with unexplained ocular changes or subtle sinus symptoms. Comprehensive imaging and endoscopic assessments are essential for revealing underlying sinus pathologies that might not be evident through physical examination alone. The diverse surgical approaches employed in these cases, ranging from sinusotomy and partial ethmoidectomy to more extensive frontal sinusotomy, highlight the importance of personalized treatment strategies tailored to each patient’s unique anatomical and pathological features. In our case, orbital floor surgery was not performed as there were no associated visual or cosmetic concerns. The favorable outcomes observed across these cases validate the effectiveness of targeted surgical interventions in managing chronic sinus conditions and their complications.

Additionally, the potential hereditary nature of SSS warrants further investigation. The familial occurrence of CMA in this case series suggests that potential genetic or environmental factors may contribute to this syndrome’s development. However, the genetic aspect of this syndrome is yet to be explored in existing research. Additional research is needed to explore these potential links and better understand the hereditary aspects of SSS, which could significantly impact diagnostic and therapeutic approaches in the future.

## Conclusions

This case series emphasizes the critical role of comprehensive evaluation and personalized treatment in managing CMA and related disorders. It also opens the door for future investigations into the genetic aspects of SSS, aiming to improve patient outcomes through more informed and targeted approaches. Our unforeseen finding suggests that our CMA/SSS patients’ family members should be evaluated despite the lack of nasal symptoms.
